# Building a Culture of Well-Being in Primary Care Resident Training Programs

**DOI:** 10.31486/toj.19.0111

**Published:** 2021

**Authors:** R. Brent Stansfield, Heidi Kenaga, Tsveti Markova

**Affiliations:** Office of Graduate Medical Education, Wayne State University School of Medicine, Detroit, MI

**Keywords:** *Burnout–professional*, *institutional management teams*, *internship and residency*

## Abstract

**Background:** Monitoring and improving resident physicians’ well-being are crucial because clinical care burdens can cause burnout, depression, and suicide. Burnout negatively affects patient care. Promoting well-being requires cultural change best achieved through a merging of institutional top-down efforts with resident and faculty bottom-up efforts.

**Methods:** The Wayne State University Office of Graduate Medical Education targeted three residency programs (52 residents) at one hospital site for wellness interventions as part of the Alliance of Independent Academic Medical Centers (AIAMC) National Initiative VI. Institution-led efforts included promotion of employee wellness resources, prioritization of wellness at administrative meetings, and program evaluation and assessment. Resident- and faculty-led efforts included the formation of wellness committees that organized events and activities and communicated with program evaluation committees to address wellness concerns. Impact was assessed using mixed methods: the quantitative Resident Wellness Scale, a modified form of the Medical School Learning Environment Survey, and a qualitative Resident Wellness Semi-Structured Interview.

**Results:** Institutional efforts were successfully applied through multiple administrative channels. Resident-led efforts were less successful initially, but wellness committees led by faculty champions were formed within programs and strengthened the resident-led efforts. Quantitative measures indicated that well-being increased and then declined, perhaps attributable to cohort effects. Qualitative analysis revealed multiple dimensions of well-being. We discuss limitations of the work and future directions.

**Conclusion:** Resident well-being requires cooperation and a combination of top-down institutional and bottom-up trainee efforts. Because resident well-being is a complex phenomenon, efforts to improve and sustain it must also be multidimensional and broadly applied.

## INTRODUCTION

Residency, the phase of medical education during which physicians train in their chosen specialty, requires long work hours with intense supervision and assessment of clinical performance, so resident well-being is a profound concern in academic medical centers. Workplace stressors and the burdens of clinical care place resident trainees at high risk of burnout, depression, and suicide.^1,2^ Resident burnout is associated with higher medical error rates and therefore impacts patient safety.^3,4^ Because of this association, the Accreditation Council for Graduate Medical Education (ACGME), the accreditation body that oversees most residency training programs in the United States, requires residency programs to monitor and improve resident well-being.^5^ In spring 2018, the ACGME began measuring residents’ well-being with a national well-being survey.

The residency learning environment is a powerful tool for reducing burnout.^6^ Systematic organizational interventions focusing on promoting well-being and resiliency, especially focusing on self-care, mindfulness, and meditation, have been shown to reduce burnout,^7,8^ and reduced work hours improve well-being.^8^ But effective strategies for improving resident well-being require more than teaching resident resilience; they require organizational change that can be difficult to implement.^6^

This sort of organizational change requires cultural change, which depends on efforts at the institutional level and by individual residents.^6,9^ Institutional leadership is necessary for providing resources, setting institutional priorities, and building infrastructure,^6^ but these efforts cannot succeed without initiative from residents and faculty. A framework for organizational change suggests that cooperation between residents and leadership is necessary for broad-based cultural change.^10^ Resident leadership is necessary because residents are motivated to improve their own wellness and can provide an accurate needs assessment,^9^ but residents cannot implement sustainable ideas without institutional resources. While the literature is clear that

organizational change is necessary to promote resident wellness, specific tactical advice on how to effect that change is less available. In this article, we describe our efforts, their measurable effects, and what we learned.

At the Wayne State University (WSU) Office of Graduate Medical Education (WSUGME), we sought to build a culture of well-being through institutional support and resident-led initiatives and to measure the impact of these changes quantitatively and qualitatively. Our project was conducted as part of National Initiative VI (NI-VI) of the Alliance of Independent Academic Medical Centers (AIAMC) at one clinical site (Ascension Providence Rochester Hospital [APRH]) that was the primary clinical site for 3 programs sponsored by WSUGME. We measured resident well-being annually through an anonymous survey from WSUGME that included the Resident Wellness Scale (RWS), a validated tool designed specifically to longitudinally track changes in the positive aspects of the well-being of medical residents.^11^ The survey also contained individual items measuring residents’ perceptions of their learning environment. Additionally, we interviewed residents to gather qualitative information about the impact of the initiative on their wellness.

## METHODS

### Participants

The project targeted 3 programs—internal medicine (36 residents), family medicine (12 residents), and transitional year (4 residents)—at APRH from fall 2017 through spring 2019.

### Timeline

The project began in November 2017. The Figure shows the timeline of the project, including institutional initiatives (top down), resident initiatives (bottom up), the formation of program-level wellness committees, and the culmination of the project in our first Professional Development Symposium in February 2019.

**Figure.**
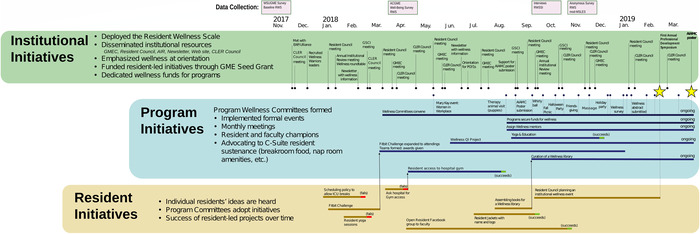
**Timeline (time plotted left to right) of wellness interventions. Institution-led initiatives are shown in the top row, program-level initiatives in the middle row, and resident-led interventions in the bottom row.** ACGME, Accreditation Council for Graduate Medical Education; AIAMC, Alliance of Independent Academic Medical Centers; AIR, annual institutional review; CLER, Clinical Learning Environment Review; EAP/Ulliance, employee assistance program run by the Ulliance company; GME, graduate medical education; GMEC, Graduate Medical Education Committee; GSCI, GMEC Subcommittee for Compliance and Improvement; ICU, intensive care unit; mod-MSLES, modified Medical School Learning Environment Survey; PGY1, postgraduate year 1 (first-year interns in residency); QI, quality improvement; RWS, Resident Wellness Scale; RWSSI, Resident Wellness Semi-Structured Interview; WSUGME, Wayne State University Graduate Medical Education Office.

### Institutional Efforts

WSUGME convened faculty and resident stakeholders to participate in our AIAMC NI-VI project (titled “Institutional and Resident-Led Wellness Interventions”) in fall 2017. Stakeholders were program directors, core faculty, and residents. They attended monthly Clinical Learning Environment Review (CLER) Council meetings chaired by the designated institutional official and with the participation of the chief medical officer, the hospital quality improvement director, and WSUGME staff members (research coordinator, director of education). This group jointly wrote the vision statement of the program: To create a sustainable culture of wellness driven by engaged, empowered residents and faculty.

Residents were contacted through email and at the CLER Council meetings and asked to generate ideas for resident wellness initiatives to be implemented on a trial basis with support from WSUGME. We promoted the AIAMC NI-VI project to the 3 target APRH programs by creating a standing agenda item at GME Committee meetings, Subcommittee for Compliance and Improvement meetings, and Resident Council meetings. Existing institutional resources (an employee assistance program, a university-based Wellness Warriors program, and an updated wellness policy) were promoted through these meetings, through the WSUGME website, and through the bimonthly WSUGME newsletter. Additionally, well-being was formalized as one of the principal focus areas of the Resident Council for the 2017-2018 academic year.

### Resident Efforts

Residents who proposed wellness activities were encouraged to find other residents to participate and to seek approval from their program directors. As shown in the Figure, resident-led efforts consisted of the following:

- Changing policy to allow for taking breaks during the intensive care unit (ICU) rotation
- Initiating a Fitbit challenge in which residents competed in teams for highest pedometer counts
- Offering yoga sessions for residents
- Allowing resident access to the hospital gym
- Inviting faculty to join a resident Facebook group
- Ordering jackets for all residents with the WSU logo
- Curating a wellness library with books, games, and puzzles
- Establishing an annual wellness event for all residents

### Wellness Committees

In spring 2018, each program convened its own wellness committee. Committees were formed without directive from WSUGME; residents’ efforts to implement their initiatives drew faculty and program director support. In each program, a faculty member less than 3 years out of residency became the faculty champion of the wellness committee. Multiple residents became active committee participants, assuming organizational and communications roles for various projects. The Figure shows the committees’ accomplishments.

### Professional Development Symposium

In February 2019, the first Professional Development Symposium was held as a capstone event. Champions of the program wellness committees organized the symposium with support from the entire Resident Council. The event was catered and included a panel discussion led by scholars and leaders in resident wellness and health, as well as information provided by local vendors and university services related to financial, physical, and dietary health. All residents, faculty, and staff were invited to attend. The event fostered a robust exchange of information and ideas and received positive feedback and the commitment to become an annual event.

### Measures

To measure the effectiveness of the intervention, we used 2 quantitative measures (the RWS and a modified form of the Medical School Learning Environment Survey) and 1 qualitative measure (the Resident Wellness Semi-Structured Interview). WSUGME obtained institutional review board approval to distribute and analyze the RWS and the Resident Wellness Semi-Structured Interview prior to data collection.

#### Resident Wellness Scale

WSUGME staff developed the RWS from a mixed methods empirical investigation in partnership with Loma Linda University to measure resident well-being.^11^ The RWS is included in the annual anonymous survey administered by WSUGME each November. It is a 10-item instrument designed to measure wellness defined as a positive construct—connectedness to meaningful work, ability, life satisfaction, institutional support, social support, and personal growth. The RWS uses a frequency scale to elicit episodic memories of thoughts and actions from the previous 3 weeks to provide a behavior-based, self-reported estimate of a resident's recent wellness level. Normative mean item responses are equal to or higher than “sometimes” (level 3 on the 5-point frequency scale) for all items.^11^

The annual anonymous survey administered by WSUGME each November measures residents’ perceptions of their programs’ learning environment (using a modified version of the Medical School Learning Environment Scale), wellness (using the RWS), curriculum, and their professional development. Additionally, the ACGME administered the RWS nationally in spring 2018 as its Well-Being Survey. The ACGME reported the results of the Well-Being Survey to programs and institutions as individual item response frequencies. We constructed a dataset combining WSUGME survey RWS responses with the ACGME Well-Being Survey responses for each item. The Figure shows the specific timepoints of these surveys.

#### Learning Environment Items

In fall 2018, the annual anonymous WSUGME survey included a modified version of the Medical Student Learning Environment Scale to measure residents’ perceptions of their learning environment. Six items were selected from the 17-item Medical Student Learning Environment Scale,^12^ a subset of a larger 55-item scale.^13^ The 17-item version is a widely used instrument for assessing medical student perception of the medical school learning environment.^14^ A consensus of 3 WSUGME staff members chose the items that pertained to aspects of the resident training learning environment vs medical school. Item wording was altered slightly to apply to residency, for example, “school” was changed to “program” and “student” was changed to “resident.” Because the construct validity of this collection of items is untested, we only analyzed the items individually and not as measures of a common construct. These learning environment items were rated on a 5-point Likert disagreement scale: 2 of the items were worded in the opposite valence of the other 4 items.

#### Resident Wellness Semi-Structured Interview

The Resident Wellness Semi-Structured Interview was developed to elicit qualitative responses to the perception and impact of all wellness events or activities that the interviewee was aware of. The interview was designed as a one-on-one semi-structured interview to elicit thoughts and ideas around residents’ participation in activities related to well-being and their impact.^15^

Purposeful sampling^16^ was used to collect interviews from residents who were typical in their usage and response to wellness activities. Selected residents were invited to participate, but participation was voluntary.

A WSUGME staff member conducted the interviews in fall 2018. The interviewer was known to the residents and had announced his departure from the office for another job which facilitated resident comfort in confiding in him.

### Analysis

#### Resident Wellness Scale Ratings Over Time

To measure changes in well-being over time, we performed a one-way analysis of variance for each of the 10 RWS items at the 3 time points (fall 2017, spring 2018, and fall 2019) treated as a categorical variable. Treating time as a categorical variable allowed testing for seasonal (fall vs spring) changes and accounting for a different population of residents in the second fall survey because of graduation and matriculation.

#### Correlation of Resident Wellness Scale With Learning Environment Items

To measure the relationship between residents’ well-being and their perception of the learning environment, Pearson correlation coefficients were computed for the 6 learning environment items with the 10 RWS items. Construct validity was not assumed; items were tested individually and not as parts of a coherent scale. Positive, statistically significant correlations between two items indicated that residents who rated one item highly also tended to rate the other highly. Correlations had 45 degrees of freedom, so correlations stronger than *r*=0.28 were statistically significant at the α=0.05 level.

#### Resident Wellness Semi-Structured Interview Analysis

A professional transcription service transcribed recorded interviews. A WSUGME administrator who was not involved in the analysis of the interviews reviewed the transcripts and removed any identifying information. Interviews were analyzed using a grounded theory approach. Two coders, the primary author and another WSUGME administrator, independently read the deidentified interview transcripts and isolated themes. The coders met to compare themes, to identify the commonalities of their readings, and to reach consensus on the themes.

All quantitative analyses were conducted using R, version 3.4.4 (R Foundation for Statistical Computing).^17^

## RESULTS

### Response Rate

At baseline in fall 2017, the response rate to the RWS on the annual anonymous WSUGME survey was 54%. On the ACGME Well-Being Survey in spring 2018, the response rate was 100%. On the final annual anonymous WSUGME survey in fall 2018, the response rate was 94%. Of 10 residents invited to be interviewed for the Resident Wellness Semi-Structured Interview, 9 agreed (90%).

### Resident Wellness Scale Ratings Over Time

Table 1 shows the results of the one-way analysis of variance models of RWS items. Baseline ratings for items were between 3.1 and 4.1. These means are comparable to published administrations of the RWS.^11^ Five items showed statistically significant time effects: item 1 (“Reflected on how your work helps make the world a better place”), item 2 (“Felt the vitality to do your work”), item 7 (“You felt your basic needs are met”), item 9 (“Knew who to call when something tragic happened at work”), and item 10 (“You felt connected to your work in a deep sense”). For most RWS items, mean ratings increased from fall 2017 to spring 2018 and then decreased in fall 2018.

**Table 1.** Mean Resident Wellness Scale Item Ratings and Changes From Baseline With One-Way Analysis of Variance Results for Each Item

Resident Wellness Scale Item	Baseline Fall 2017^a^	Spring 2018^b^	Fall 2018^a^	*F* Test	*P* Value
1. Reflected on how your work helps make the world a better place	3.1	3.7 (+0.62)	3.2 (+0.04)	*F*_(2,142)_=6.63	**0.0018**
2. Felt the vitality to do your work	3.6	4.0 (+0.43)	3.7 (+0.08)	*F*_(2,143)_=3.37	**0.0371**
3. Felt supported by your coworkers	3.8	4.0 (+0.19)	4.0 (+0.20)	*F*_(2,143)_=0.60	0.5529
4. Had an enjoyable interaction with a patient	4.1	4.4 (+0.34)	4.2 (+0.12)	*F*_(2,143)_=2.98	0.0537
5. Was proud of the work you did	4.0	4.1 (+0.11)	3.9 (–0.07)	*F*_(2,142)_=0.61	0.5446
6. Was eager to come back to work the next day	3.5	3.7 (+0.21)	3.2 (–0.20)	*F*_(2,143)_=2.25	0.1086
7. You felt your basic needs are met	3.7	4.1 (+0.42)	3.8 (+0.11)	*F*_(2,142)_=3.61	**0.0296**
8. You ate well	3.7	4.0 (+0.34)	3.7 (–0.01)	*F*_(2,142)_=2.71	0.0699
9. Knew who to call when something tragic happened at work	3.8	4.4 (+0.64)	3.8 (–0.05)	*F*_(2,140)_=11.90	**0.0000**
10. You felt connected to your work in a deep sense	3.9	3.9 (–0.01)	3.3 (–0.64)	*F*_(2,143)_=7.66	**0.0007**

^a^Administered by the Wayne State University Office of Graduate Medical Education.

^b^Administered by the Accreditation Council for Graduate Medical Education.

Note: Significant *P* values (*P*≤0.05) are in bold type.

### Correlations of Resident Wellness Scale and Learning Environment Items

Table 2 shows Pearson correlation coefficients for each RWS item with each learning environment item from the anonymous annual WSUGME survey in fall 2018. Correlations showed 3 meaningful patterns. First, learning environment items measuring disagreement about a distance between residents and faculty (“I often hesitate to express my opinions and ideas to faculty or my Program Director” and “Faculty are reserved and distant with residents”) correlated positively with RWS items measuring vitality, eagerness to come back to work, having basic needs met, and deep connectedness to work. Second, learning environment items measuring disagreement about openness and trust in the program (“Upper-level residents provide support and guidance to junior residents” and “My program fosters an environment of mutual trust and respect among residents, faculty, patients, nurses, and staff”) correlated negatively with most RWS items. Third, learning environment items measuring disagreement that the program is responsive to needs (“Resident complaints are responded to with meaningful action” and “Faculty, administrators, and staff give personal help to residents having academic difficulty”) showed weaker correlations with RWS items.

**Table 2.** **Pearson Correlation Coefficients Between Resident Wellness Scale and Learning Environment Items (Fall 2018)**

	Learning Environment Item					
Resident Wellness Scale Item	I often hesitate to express my opinions and ideas to faculty or my Program Director.	Resident complaints are responded to with meaningful action.	Faculty are reserved and distant with residents.	Faculty, administrators, and staff give personal help to residents having academic difficulty.	Upper-level residents provide support and guidance to junior residents.	My program fosters an environment of mutual trust and respect among residents, faculty, patients, nurses, and staff.
1. Reflected on how your work helps make the world a better place	0.22	–0.14	0.14	0.09	**–0.39**	–0.24
2. Felt the vitality to do your work	**0.43**	–0.22	**0.36**	–0.08	**–0.29**	–0.40
3. Felt supported by your coworkers	**0.28**	–0.20	0.18	–0.16	**–0.37**	**–0.32**
4. Had an enjoyable interaction with a patient	0.19	–0.10	**0.35**	–0.02	0.15	**–0.37**
5. Was proud of the work you did	0.23	–0.18	0.18	0.00	**–0.28**	**–0.37**
6. Was eager to come back to work the next day	**0.31**	–0.19	**0.42**	–0.14	**–0.33**	**–0.47**
7. You felt your basic needs are met	**0.39**	–0.06	**0.38**	–0.21	–0.18	**–0.34**
8. You ate well	**0.45**	–0.16	0.26	**–0.33**	**–0.42**	–0.27
9. Knew who to call when something tragic happened at work	0.27	–0.06	**0.29**	–0.13	**–0.47**	**–0.41**
10. You felt connected to your work in a deep sense	**0.31**	–0.19	**0.28**	–0.02	**–0.29**	**–0.44**

Note: Significant correlations are in bold type.

### Resident Wellness Semi-Structured Interview Results

The interviews revealed at least 3 common themes. First, residents valued participation in the activities involving groups of residents (eg, Fitbit challenge, yoga sessions, wellness committee events such as the Mary Kay visit, and therapy animals). Activities that occurred at the hospital during shifts (eg, yoga session, Mary Kay visit, and therapy animals) were valued because they were well attended (“Everybody participated. I think that was a big part of why it was enjoyable. Everybody seemed to enjoy it and come in and out of the room.”). Second, residents found value in the anticipation of wellness activities. Many residents remarked on the positive value of the anticipation (“I think half the fun is actually having something to look forward to, not exactly the direct impact.” “Everybody seems very excited. Almost everybody RSVP’d already. That’ll be fun.”). Third, regular contact with friends and family outside of work was a common way of maintaining wellness (in response to the interview question, “What do you do when things get tough at work?” a resident responded, “Call my family, support network outside of the residency program.”).

Some residents mentioned additional aspects of wellness that they felt should be addressed by the institution (for example, “…others have issues like financial issues or like immigration issues.”). Such comments reflected implicit concern that wellness events tended to focus on relaxation, leisure, and food. Some residents explicitly noted that other aspects of well-being, such as meaning and purpose in work and life, needed more emphasis (“A party's good, but sometimes, maybe, if you have stress outside the hospital or have personal stress, even, you’ll not enjoy the parties or all this stuff because already you have your own problem.”). Some residents noted that wellness events were perceived as intrusive into the workday (“If it was a really busy day in the middle of the week, it probably wouldn’t have been enjoyed as much ’cause we have so many other things that we need to be doing.”). Residents often mentioned their proximity to their immediate family or spouse was important for their ability to maintain well-being.

## DISCUSSION

Between fall 2017 and fall 2018, WSUGME led a concerted effort to build a culture of wellness in 3 APRH residency programs. The efforts were bottom-up (resident-led initiatives) and top-down (institutional pushing of resources and focus) and were best realized at the convergence of these efforts through wellness committees, groups of faculty champions and residents who met regularly. This union of top-down and bottom-up efforts mirrors descriptions of convergence in the leadership literature, for instance, “the joining and/or combining of top-down efforts led by those in positions of authority and bottom-up efforts led by those without positions of authority.”^10^

Significant positive changes were observed for 3 of the 10 RWS items (“Reflected on how your work helps make the world a better place,” “Felt the vitality to do your work,” and “You felt your basic needs are met”) and a significant decline for 2 items (“Knew who to call when something tragic happened at work” and “You felt connected to your work in a deep sense”). The RWS is designed to measure changes in wellness above the more commonly measured cut points for burnout and depression^11^ and thus captures a wide spectrum of wellness differences. The increase in RWS item ratings from baseline to the second timepoint and subsequent decrease in ratings at the third timepoint could be attributable to an upward bias of respondents to the ACGME survey because they understand that the ACGME is an accrediting authority. Other researchers have seen this bias.^18^ Another important note is that senior residents who benefitted from the wellness interventions graduated from their programs in June, and new interns who had not yet been exposed to those interventions arrived in July. This rotation could also explain why item rating means were higher in the spring but lower the following fall (Table 1). Because education is cyclical, structural interventions such as wellness committees are more likely to be sustainable and effective compared to interventions that target individual residents.

Resident-led initiatives were implemented concurrently and met with varying levels of success. For instance, a change in policy to allow for taking breaks during the ICU rotation was abandoned because of administrative pushback. The Fitbit challenge was well received and expanded to include faculty and staff. Yoga sessions for residents were sparsely attended and abandoned. Resident access to the hospital gym failed as a resident-led initiative but was successful when the program leadership became involved and discussed the gym access policy with hospital staff. Inviting faculty to join a resident Facebook group allowed casual contact between residents and some faculty. Ordering jackets for all residents with the WSU logo was very successful, with residents wearing their jackets around the hospital. Curating a wellness library with books, games, and puzzles was expanded to include dedicated weekly puzzle time activities. Establishment of a wellness event for all residents succeeded and became an annual event: the Annual Professional Development Symposium.

Committee-led initiatives were more successful and persisted longer than resident-led initiatives. Wellness committees organized monthly get-together activities outside of work hours that were attended by more than half of residents. Committees organized special events during work hours such as Puppies and Ice Cream (an animal shelter brought puppies to play with and the program provided ice cream), a massage session (a masseuse gave free 10-minute massages), and a cosmetician-led Mary Kay makeup demonstration for female residents.

The results of the Resident Wellness Semi-Structured Interview analysis suggest that wellness events are valued because of the social interaction (high participation in the workplace) and that social connections outside of work are also an important aspect of resident well-being. Residents reported enjoying the anticipation of events, although some noted that events could be disruptive and did not address all sources of resident stress. As should be evident from this research, resident well-being is a complex phenomenon and therefore most likely requires a complex intervention to improve it.

This study has several limitations. The initiative was performed at only one clinical site and among primary care programs, so the findings may not generalize to other sites or to other specialties. WSUGME will apply the lessons learned from this project to our other programs and sites and will continue to evaluate the efficacy of those interventions. Our quantitative data have sampling bias: a low response rate at baseline and the possibility of an upward response bias to the ACGME survey. Replication of quantitative trends observed here is needed. Because the interventions were concurrent, reliably estimating their individual impact on resident well-being is not possible. Our conclusions are therefore limited to noting the relation between the learning environment and resident well-being, showing the importance of merging bottom-up and top-down intervention strategies, and describing how individuals’ different perspectives relate to the multifaceted nature of resident well-being.

## CONCLUSION

WSUGME will continue to underscore the centrality of resident wellness and professional development as an institutional initiative. APRH residency programs in other specialties at other hospital sites have formed wellness committees that WSUGME will support by requesting updates at bimonthly GME Committee and Resident Council meetings. We will continue to use the RWS at the program level to track changes longitudinally.

## Notes

This article meets the Accreditation Council for Graduate Medical Education and the American Board of Medical Specialties Maintenance of Certification competencies for Medical Knowledge, Interpersonal and Communication Skills, Systems-Based Practice, and Practice-Based Learning and Improvement.
